# SunSmart Accreditation and Use of a Professional Policy Drafting Service: Both Positively and Independently Associated with High Sun Protective Hat Scores Derived from Primary School Policies

**DOI:** 10.1155/2020/9695080

**Published:** 2020-06-30

**Authors:** A. I. Reeder, E. E. Iosua, B. McNoe, A.-C. L. Petersen

**Affiliations:** ^1^Social & Behavioural Research Unit, Department of Preventive & Social Medicine, Dunedin School of Medicine, University of Otago, Dunedin, New Zealand; ^2^Centre for Biostatistics, Division of Health Sciences, University of Otago, Dunedin, New Zealand

## Abstract

**Background:**

The head and neck are exposed to the highest solar ultraviolet radiation levels and experience a disproportionate skin cancer burden. Sun protective hats can provide an effective barrier. Since early life exposure contributes to skin cancer risk, the World Health Organisation recommends prevention programmes in schools. The New Zealand SunSmart Schools programme is one example. Two criteria concern wearing hat outdoors: students are required to wear a hat providing protection for the face, neck, and ears; if a suitable hat is not worn, students must play in shaded areas.

**Objectives:**

To investigate two internationally relevant interventions as plausible statistical predictors of hat policy strength: (1) skin cancer primary prevention programme membership, (2) use of a professional policy drafting service.

**Methods:**

Of 1,242 (62%) eligible schools participating in a 2017 national survey, 1,137 reported a sun protection policy and 842 were available for categorising and allocating protective scores (0–3).

**Results:**

In multinomial (polytomous) logistic regression models of cross-sectional association, adjusted for school characteristics, SunSmart accredited schools and those utilising a policy drafting service were independently significantly more likely than their counterparts to obtain the most protective compared to the least protective hat score (respectively, RRR 6.48: 95% CI 3.66, 11.47; 7.47: 3.67, and 15.20). For the dichotomous shade measure, similar associations were found using adjusted logistic regression (OR 3.28: 95% CI 2.11, 5.09; 2.70: 1.54, 4.74).

**Conclusions:**

Our findings provide support for two plausible interventions that could potentially be implemented beneficially in primary schools via established infrastructure in any jurisdiction, internationally.

## 1. Introduction

The head and neck are the body areas exposed to the most solar ultraviolet radiation (UVR), while the face and neck are 2–4 times more sun sensitive than the limbs [1]. These are the areas where most keratinocyte skin cancers occur and, with the head and neck skin surface approximately only 9% of total body area, a disproportionately higher density of cutaneous melanomas than other body sites [1]. Most of these melanomas tend to be located on the face (as much as 60.4% in one study), followed by the scalp (24.9%) and neck (14.7%).

Head and neck melanomas have a poorer prognosis than cutaneous melanomas at other sites [2]. In a population-based study, location on the scalp and neck was an independent adverse prognostic factor of disease-specific survival in multivariable analysis [3]. The physical location of these lesions makes them liable to disease progression because they are sometimes not readily identifiable by self-inspection, being hidden under hair, within or behind the crevices of ears, or on the back of the neck, and delayed detection and treatment contributing to poor prognosis. Surgical treatment in these areas can also be invasive and highly visible to others.

Accordingly, sun protective hat wearing is an important skin cancer primary prevention strategy which also provides increased protection against ocular UVR damage. A suitable sun protective hat provides complete protection for the scalp and an effective barrier against most direct UVR reaching the face, neck, and ears [4]. This protective coverage is more certain and sustained than that provided by sunscreen; however, sites other than the scalp remain subject to reflected UVR and may require supplementary protection.

In view of their relatively greater frequency and poorer prognosis, but potentially greater preventability by the physical barrier of a suitably sun protective hat, the head and the neck deserve greater attention for primary prevention. Worldwide, sun exposure is estimated to cause about 65% of cutaneous melanomas, but in regions where high sun exposure occurs, as much as 95% of melanomas and 99% of keratinocyte skin cancers are considered potentially preventable through the avoidance of exposure to harmful UVR levels [5].

Since early life UVR exposure and sun protection practices contribute to subsequent skin cancer risk, [6] it is particularly appropriate that schools follow evidence-based, skin cancer prevention programmes, as recommended by the World Health Organisation [7]. There is evidence that schools with policies that promote primary skin cancer prevention have superior sun protective practices [8]. This may be because the drafting of a sun protection policy acknowledges that a need exists to address the issue of skin cancer primary prevention and implies a commitment to action and the seeking of practical solutions. Furthermore, once implemented, such policies provide opportunities to monitor the compliance and hold the institutions to account.

## 2. Objectives

Our main study objectives were as follows. While controlling for school demographic factors, to investigate two internationally relevant, potentially relatively easily implemented interventions that are plausible potential statistical predictors of the strength of school sun protective hat policies, namely, (1) membership of a skin cancer primary prevention programme and (2) use of a professional policy drafting service.

## 3. Materials and Methods

### 3.1. Study Design

We used multivariable modelling of comprehensive cross-sectional New Zealand (NZ) data sourced from the Ministry of Education, [9] the NZ SunSmart Schools Accreditation Program (SSAP), and the 2017 national school survey which was implemented to help evaluate that program [10].

### 3.2. Setting

Under an administratively devolved educational system, each school's Board of Trustees is responsible for drafting a range of policies, including those relating to sun protection, although the latter is not compulsory. Participation in the SSAP, nationally implemented in 2005 by the Cancer Society of New Zealand, is voluntary and promoted to schools with a student age range of approximately 5–12 years [11, 12]. Sun protection policies operate primarily during terms 1 and 4, when antipodean solar UVR levels are highest. The SSAP is based on meeting 12 criteria. In summary and in addition to the two-student hat wearing criteria (1 and 2), these include that the school has to have a sun protection policy (3) about which all of the school community must be informed (4). The wearing of sun protective clothing (5) and broad spectrum sunscreen of at least SPF30 (6) is encouraged. School staff are expected to role model the wearing of sun protective hats (7). Sun protection is routinely considered in formal planning documents for outdoor events (8) and activities are rescheduled whenever possible to minimize time spent outdoors during high UVR times (9). The school should either have sufficient shade or be working towards this for most passive outdoor activities (for example, eating lunch and outdoor classes) (10) and the school Board reviews the shade policy at least triannually (11). In addition, SunSmart education should be included in the curriculum (12).

### 3.3. Procedures

Policy documents were sought from the 1,242 schools that participated in the 2017 national survey about sun protection in all schools attended by primary level students [10]. Of these, 1,137 reported having a sun protection policy and 842 policies were available for inspection. Seven schools were included which were found to have a policy but had not reported that. Some (*n* = 22) Māori (the indigenous population of NZ) and total immersion language schools (Kura) responded to the national survey but are not included in this study because of their small numbers but large differences across a broad range of demographic factors and because these schools carry out all of their education and instruction in the Māori language, whereas the SSAP is, at present, only available in English.

### 3.4. Measures

#### 3.4.1. Outcome Variables

Interrater reliability (ACLP and AIR) for scoring the outcome variables was initially tested against 20 sequential policies after which adjustments were made to clarify definitions. Reliability for the full study, using the revised criteria and tested between two researchers (BM and AIR) against another 100 randomly selected policies, was 93%. Of the remaining 7% of scores, those clearly in error were then corrected before analysis.

#### 3.4.2. Hat Wearing

When outdoors during breaks, lunchtimes, excursions, or similar activities, students are required to wear a suitably sun protective hat that provides protection for the face, neck, ears, and eyes [4]. Hats were categorised and allocated a protective score based on SSAP criteria. Policies meeting criteria for both specified hat type(s) and dimensions were allocated the highest score (i.e., 3). Hat types included three options: “broad brimmed (minimum 7.5 cm brim), Legionnaires', or bucket hats (minimum 6 cm brim, deep crown).” Dimensions were not specified for Legionnaires' hats. Policies which specified that either a “sun protective” or “sun” hat was required, but specific dimensions were not available, were allocated a score of 2. Policies that mentioned the need to wear a hat when outdoors, but lacked sun protective specificity, were allocated a score of 1. A score of zero was allocated when the policy either did not mention the requirement to wear a hat when outdoors or permitted the wearing of a “cap,” whatever other requirements were described. Caps are not acceptable for SunSmart accreditation because they do not provide protection for the sides of the face, including the neck and ears [13]. An exception was only made for specific sports participation where peripheral vision is required and so a sun protective hat may not be appropriate, but these cases caps had to be worn in conjunction with the application of complementary facial sunscreen.

#### 3.4.3. Shade Use Requirement

The second hat-related SSAP criterion specifies that if a hat is not worn then that student is required to play in a shaded area. This was scored dichotomously as either 1 (met the criterion) or 0 (failed to meet the criterion).

#### 3.4.4. Statistical Predictor Variables

Two independent dichotomous variables were investigated as potential statistical predictors of the two policy outcome variables. These variables were as follows: (a) SunSmart status (accredited or not accredited), and (b) whether or not the school had used a professional agency (hereafter designated SchoolDocs) to help draft sun protection policy [14]. The first of these variables was obtained from the Cancer Society national SSAP database and approximately 37% of eligible schools were accredited, thereby ensuring reasonable variation for analysis. The second variable was obtained from responses to the 2017 national school survey. [10]. For a fixed annual subscription based on school roll size, ranging from $1,100–$2,500 (2017 subscription, excluding 15% Goods and Services Tax), the SchoolDocs commercial agency provides schools with policy templates, including for sun protection, which can be modified to meet specific school needs and requirements.

#### 3.4.5. Potential Confounders

Variables considered controlling for in the multivariable modelling were comparable to those used for an earlier study [12] and similarly obtained from the Ministry of Education database. Following the listing order in Table 1, the first variable documented was whether schools were state, state-integrated, private, or partnership institutions. State schools represent the largest group and are publically funded through the state educational budget. State-integrated schools are former private schools which have chosen to accept the public funding and be integrated into the state education system while retaining their “special character,” such as administration through a religious faith or using specialist education methods, e.g., Steiner or Montessori. Private schools are those that have remained independent and are largely funded through fees paid by parents. School socioeconomic decile rating ranged upward from 1, the lowest, which included the 10% of schools with the highest proportions of students enrolled from low socioeconomic communities. School type included full primary with students 5–13 years of age, Contributing (5–11 years), Intermediate (10–13 years), and Composite schools which are institutions that additionally include various levels of secondary classes. A dichotomous variable was initially created to distinguish schools with primary students, alone, from those with both primary and secondary students. Schools were also categorised by roll numbers into four groups: very small, small, medium, and large size by geographic region (the six Cancer Society regional divisions used for health promotion dissemination and useful for monitoring advocacy efforts and institutional change) and by four Ministry of Education population density categories (rural, minor urban, secondary urban, and main urban). Almost all schools were coeducational.

### 3.5. Analysis

The analyses included most of the variables listed in Table 1, but excluded from modelling were the 14 private schools, both of the “school type” variables and the “gender” variable because, in each case, of small numbers in particular categories.

The chi-squared goodness-of-fit test was used to assess the representativeness of participating schools in terms of school socioeconomic characteristics (Table 1). Monte Carlo exact tests were alternatively used for expected cell counts <5. Associations between the hat score (0–3) and both SunSmart accreditation status and use of the SchoolDocs service were investigated using unadjusted and adjusted multinomial (polytomous) logistic regression to provide relative risk ratios (RRR) and 95% confidence intervals. Identical associations, but with the compulsory shade outcome measure, were investigated using unadjusted and adjusted logistic regression. The initial unadjusted models included the accreditation and SchoolDocs variables only, whereas, to control for potential confounding, the multiple regression models additionally incorporated the remaining school characteristics. Variance inflation factors were investigated for collinearity. Stata software version 14.2 was used for all analyses, and the two-sided significance level *α* = 0.05 was specified for all statistical tests.

## 4. Results

Survey responses were received from 1242 (62%) eligible schools, and their distribution according to the characteristics was recorded in the Ministry of Education database, and the policy documentation obtained is presented in Table 1. Responding schools were broadly representative, but SunSmart-accredited schools were somewhat over represented. Schools for which policy documents were obtained were, broadly, comparable to schools which had a policy for assessment (842/1137) across all sociodemographic factors, except geographic region (where there were more from the Waikato/Bay of Plenty and Canterbury/West Coast Divisions and less from Northland/Auckland than expected) and accreditation status (with more accredited schools than expected). Overall, 202 (24%) schools reported using the SchoolDocs service.

### 4.1. Hat and Shade Scores

Less than half of the schools for which policy documents could be obtained met the optimum score for hat type (Table 2). Overall, 90 schools had policies that only recommended (or encouraged, requested etc.), rather than required, hat wearing was included among those conservatively scored as zero. Schools where the wearing of caps was permitted either in general (*n* = 10) or under specified circumstances, but without being complemented by the requirement to wear facial sunscreen (*n* = 1), were also similarly conservatively scored. An exception was made in one case for specific sports participation where peripheral vision was required and so a fully sun protective hat was not appropriate, but it was specified that a cap had to be worn in conjunction with application of complementary facial sunscreen.

The policies of more than 80% of schools met the criterion for specifying shade use when a hat was not worn (Table 2). The suggestion of negative consequences for not wearing a hat was indicated in 66 school policies, for example, “no hat, no play” rather than “no hat, play in the shade.” Evidence of positive reinforcement for hat wearing was lacking.

### 4.2. Statistical Analysis

With respect to the two dichotomous potential statistical predictors of interest, SSAP status and the use of a professional policy drafting service (SchoolDocs), in Table 3, we report our investigation of any associations between these factors and both the strength of hat wearing scores and shade score derived from policy documents.

Inferences between the unadjusted and adjusted models were analogous for both accreditation status and SchoolDocs. Accredited schools were generally associated with an increased relative risk in achieving higher hat scores compared with a lower score (Table 3). Specifically, they were significantly more likely to obtain a high score of 3 or 2 rather than any lower score. The positive association between use of the SchoolDocs service and hat score was less consistent, with those schools employing the service, only more likely to achieve the highest score compared with either a score of 0, 1, or 2. There was no evident difference in the relative risk of scoring a 2 compared to a 0 or 1, or a 1 compared to a 0.

The unadjusted and adjusted logistic regression models also produced similar inferences in the shade analyses. The odds of an accredited school prescribing compulsory shade was 3.28 (CI: 2.11–5.09) times the odds of an unaccredited school. This represents a 228% increase in the odds. Correspondingly, use of SchoolDocs was also associated with an increased odds of incorporating a shade requirement in the school's sun protection policy (OR: 2.70; CI: 1.54–4.74).

## 5. Discussion and Conclusions

Our study demonstrates that, despite its potential significance for the prevention of head and neck skin cancers, there remains a substantial scope for improvement in NZ primary school sun protection hat wearing policy, with only 43% meeting optimal criteria. This compares to the finding that 62% of school districts in Colorado and California had a sun protection policy for hats with a brim, [15] although only 32.5% of school principals in California were aware of this [16]. Probably, the highest rates (89%) have been reported for hat wearing expectations in policies in Queensland, Australia [17].

Three of our study findings are relevant for potentially improving this situation. First, we found that SunSmart-accredited schools were significantly more likely than their nonaccredited counterparts to obtain higher and more protective scores for hat wearing and shade use policies. This positive association between membership of an organised school sun protection program and the strength of hat wearing and shade use policies suggests that such programs may help improve the policy by requiring the schools to meet the minimum criteria when applying for accreditation. This is compatible with an Australian finding that SunSmart membership was indirectly related to practice comprehensiveness via policy comprehensiveness [18]. Anecdotally, the Cancer Society of NZ health promotion staff who administer the SSAP have commented that these policies provide continuity when school staff changes occur, in particular, when a principal is replaced and commitment to the SSAP may slip. Potentially attractive programme characteristics include that it is free and provides the opportunity to promote the school as being accredited and a socially responsible, safe place for students and the whole school community. Once accredited, schools receive a SunSmart Schools Accreditation Certificate, a sign for the school building or gate, and a media release for the local newspaper.

Second, schools which utilised a professional drafting service for policy development, were more likely to have higher scores than those that did not. This suggests that such services should be encouraged because they may help strengthen policy as well as potentially ensure a more comprehensive, unambiguous specification of all recommended criteria and encourage consistency between schools. There is evidence that a comprehensive written policy is associated with better and more comprehensive sun protection practices [8, 18]. Currently in NZ, schools have to pay an annual fee for this drafting service, from a minimum of $1,100 for the smallest schools (50 students or fewer) up to $2,500 for large schools (2,400 or more students). Although this cost is not large when viewed in the context of total school expenditure, it may be a barrier to uptake. However, since the fee covers all policies, not just those for sun protection, it significantly reduces the need for schools to commit resources to policy development. Comprehensiveness, consistency, and equity in sun protection are likely to be promoted if the NZ Ministry of Education or the equivalent agencies in other jurisdictions, internationally, were to meet the cost of such a service.

Third, each of the two key statistical predictors, both SunSmart school accreditation and use of a policy drafting service, produced independent positive associations, because both factors were included concurrently in all models. Although a few isolated, statistically significant relationships between school socioeconomic characteristics and the outcomes of interest were found in the initial modelling, these did not follow any discernibly consistent patterns, were difficult to interpret and, in contrast to the two key predictors, are likely to be less readily targeted or modifiable through the implementation of skin cancer primary prevention interventions. An Australian finding that the relationship between written policy and practice was stronger for remote and regional schools is of interest in this context [18] but would require confirmation in onsite studies and, in the present study, no consistent relations between such school characteristics were found with respect to the written policy, alone.

Our study had some limitations. First, although the overall response rate (62%) was acceptable and responding schools did not differ substantially from nonresponders with respect to known socioeconomic factors, those schools for which policies were available for analysis were less representative. Accordingly, it is likely that the study findings reflect a positive response bias, for example, because policies were more likely to be available for accredited than nonaccredited schools. Second, our findings are based on analysis of policy documentation rather than the onsite observation. Actual practice is likely to be less protective than the available data suggest, so onsite studies would be valuable to confirm the differences in practice. Third, since the study was cross-sectional in design, the statistical predictors and outcome variables were not subject to temporal separation, so causation cannot be attributed.

Despite these limitations, our study findings provide support for two relatively straightforward, plausible interventions that could potentially be implemented beneficially in all primary schools via established administrative infrastructure in any jurisdiction, internationally. In both Australia and NZ, such school programmes have been reinforced by broad social marketing mass media campaigns to raise awareness of skin cancer prevention as well as interventions in workplaces and recreational settings. The Australian evidence, in particular, demonstrates that social marketing can be effective not only in motivating behaviour change, reducing sunburn, and increasing awareness but also reducing melanoma rates and having positive economic effects [19]. However, this requires long-term investment or any positive changes may quickly be eroded. School programmes are an important component of this broader strategy.

## Figures and Tables

**Table 1:** Distribution of school characteristics by response status.

School characteristics	All eligible schools (*N* = 2,010)		Responding schools (*N* = 1,242)		Policy (*N* = 1,137)		Policy accessed (*N* = 842)	
	*n*	%	*n*	%	*n*	%	*n*	%
Integration status								
Partnership	4	0.20	2	0.16	0	0.00	0	0.00
Private	69	3.43	40	3.22	29	2.55	14	1.66
State	1,678	83.48	1,039	83.66	967	85.05	734	87.17
State-integrated	259	12.89	161	12.96	141	12.40	94	11.16
Socioeconomic decile								
1 (lowest)–3 (low)	559	27.81	316	25.44	281	24.71	206	24.47
4–7 (medium)	768	38.21	479	38.57	452	39.75	333	39.55
8(high)–10 (highest)	654	32.54	431	34.70	394	34.65	299	35.51
Missing data	29	1.44	16	1.29	10	0.88	4	0.48
Type								
Composite (1–10 years)	2	0.10	2	0.16	1	0.09	0	0.00
Composite (1–15 years)	113	5.62	68	5.48	51	4.49	30	3.56
Restricted composite (7–10 years)	5	0.25	3	0.24	2	0.18	1	0.12
Contributing (1–6 years)	761	37.86	488	39.29	459	40.37	356	42.28
Full primary (1–8 years)	1,012	50.35	615	49.52	572	50.31	432	51.31
Intermediate (7–8 years)	117	5.82	66	5.31	52	4.57	23	2.73
Type (dichotomised)								
Primary and secondary	120	5.97	73	5.88	54	4.75	31	3.68
Primary	1890	94.03	1169	94.12	1083	95.25	811	96.32
Overall roll size								
Less than 51	296	14.73	166	13.37	150	13.19	112	13.30
51–200	706	35.12	459	36.96	423	37.20	328	38.95
201–400	549	27.31	337	27.13	309	27.18	226	26.84
Greater than 400	452	22.49	275	22.14	251	22.08	175	20.78
Missing data	7	0.35	5	0.40	4	0.35	1	0.12
Gender status								
Single sex (girls)	10	0.50	8	0.64	4	0.35	2	0.24
Single sex (boys)	9	0.45	7	0.56	5	0.44	2	0.24
Coeducational	1,991	99.05	1,227	98.79	1,128	99.21	838	99.52
Geographic region (N to S)^a^								
Northland/Auckland	564	28.06	333	26.81	291	25.59	175	20.78
Waikato/Bay of Plenty	355	17.66	213	17.15	203	17.85	180	21.38
Central Districts	343	17.06	198	15.94	179	15.74	119	14.13
Wellington/Tasman	283	14.08	189	15.22	170	14.95	126	14.96
Canterbury/West Coast	272	13.53	185	14.90	180	15.83	154	18.29
Otago/Southland	193	9.60	124	9.98	114	10.03	88	10.45
Population density status^b^								
Rural (<1,000)	616	30.65	378	30.43	351	30.87	268	31.83
Minor urban (1,000–9,999)	216	10.75	139	11.19	129	11.35	107	12.71
Secondary urban (10,000–30,000)	112	5.57	75	6.04	67	5.89	60	7.13
Main urban (>30,000)	1,061	52.79	647	52.09	588	51.72	406	48.22
Missing data	5	0.25	3	0.24	2	0.18	1	0.12
Accredited								
Yes	826	41.09	562	45.25	551	48.46	527	62.59
No	1,184	58.91	680	54.75	586	51.54	315	37.41

^a^ Cancer Society Divisions. ^b^ Ministry of Education categories.

**Table 2:** Numbers and percentages of schools with hat and shade scores.

Score components (highest = best)	Schools (*n* = 842)	
	*N*	%
Hat score		
0	113	13.42
1	83	9.86
2	286	33.97
3	360	42.76

Play in shade score		
0	149	17.70
1	693	82.30

**Table 3:** Predictors of hat score from multinomial, polytomous logistic regression and meeting of shade requirement from logistic regression.

School characteristic	Hat score highest (3) vs lowest (0)		Hat score 2 vs 0		Hat score 1 vs 0		Shade requirement met	
	RRR (95% CI)	*p* value	RRR (95% CI)	*p* value	RRR (95% CI)	*p* value	OR (95% CI)	*p* value
*Unadjusted models*								
Accreditation status								
Not accredited	1		1		1		1	
Accredited	4.82 (2.99, 7.79)	<0.001	1.88 (1.20, 2.94)	0.006	0.63 (0.35, 1.31)	0.121	3.20 (2.21, 4.64)	<0.001
School documents								
No policy template	1		1		1		1	
Policy template	8.68 (4.44, 16.96)	<0.001	1.28 (0.62, 2.61)	0.505	1.65 (0.72, 3.78)	0.241	3.11 (1.84, 5.26)	<0.001

*Adjusted models* ^*a*^								
Accreditation status								
Not accredited	1		1		1		1	
Accredited	6.48 (3.66, 11.47)	<0.001	3.14 (1.82, 5.42)	<0.001	1.06 (0.52, 2.17)	0.865	3.28 (2.11, 5.09)	<0.001
School documents								
No policy template	1		1		1		1	
Policy template	7.47 (3.67, 15.20)	<0.001	1.32 (0.62, 2.81)	0.478	1.72 (0.71, 4.21)	0.231	2.70 (1.54, 4.74)	0.001

^a^Model adjusted for the school socioeconomic variables derived from Table 1 data, as described in the Section 3.5, namely, integration status, socioeconomic decile, overall roll size, geographic region, and population density.

## Data Availability

The data used to support this study can be made available from the corresponding author upon request.

## Additional Points

What is known? (i) The head and neck experience a disproportionate skin cancer burden from solar ultraviolet radiation (UVR) exposure. (ii) Early life sun exposure is linked with subsequent skin cancer risk. (iii) Sun protective hats can provide an effective barrier against solar UVR. What is new? (i) SunSmart schools programme membership and use of a professional policy drafting service are both significantly associated with the most sun protective student hat policies. (ii) These two relatively easily implemented strategies could be applied internationally.
